# P-703. Manifestations of parainfluenza type 4 infections and viral loads in children

**DOI:** 10.1093/ofid/ofae631.899

**Published:** 2025-01-29

**Authors:** Hanako Funakoshi, Meiwa Shibata, Tomoyuki Tame, Kazue kinoshita, Osamu Saitou, Yuho Horikoshi, Kenta Kuruma

**Affiliations:** Division of Infectious Diseases, Department of Pediatrics, Tokyo Metropolitan Children’s Medical Center, Fuchu sity, Tokyo, Japan; Division of Infectious Diseases, Department of Pediatrics, Tokyo Metropolitan Children’s Medical Center, Tokyo, Japan., Fuchu, Tokyo, Japan; Division of Laboratory Tokyo Metropolitan Children’s Medical Center, Futyu, Tokyo, Japan; Division of Molecular Laboratory,Tokyo Metropolitan Children’s Medical Center, Futyu, Tokyo, Japan; Department of Critical Care and Emergency Medicine, Tokyo Metropolitan Children's Medical Center, Futyu, Tokyo, Japan; Division of Infectious Diseases, Department of Pediatrics, Tokyo Metropolitan Children's Medical Center, Fuchu, Tokyo, Japan; Division of Infectious Diseases, Tokyo Metropolitan Children’s Medical Center, Tama city, Tokyo, Japan

## Abstract

**Background:**

Although parainfluenza virus type 4 (PIV-4) typically causes mild respiratory illnesses in children, it can cause severe diseases occasionally. It is not known Whether viral load of PIV-4 could correlate with severity. This study aimed to assess the relationship between PIV-4 viral load and disease’s severity in children.

Viral load and severity

**Figure:**
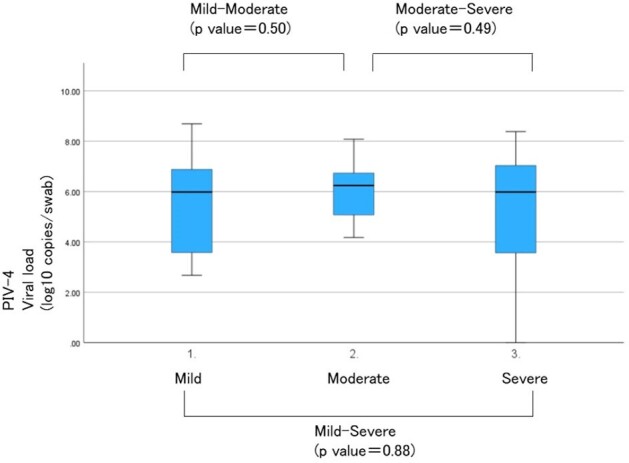

This figure shows the relationship between severity of illness and viral load.There was no correlation between viral load and severity.

**Methods:**

A retrospective study at Tokyo Metropolitan Children's Medical Center from April to October 2023 screened hospitalized children using multiplex PCR. PIV-4 positive samples were assayed for viral load of all detected viruses using real-time PCR. Cases with PIV-4 were included if PIV-4 was only detected or the viral load of PIV-4 was higher than other co-detected viruses. Demographic data, underlying diseases, laboratory, co-infections, diagnoses, and outcomes were collected from electrical charts. Cases with PIV-4 were categorized into mild cases with no oxygen demand, moderate cases with low-flow oxygen demand and severe cases with high flow nasal cannula oxygen or non-invasive/invasive mechanical ventilation or PICU admission.The viral loads of PIV-4 and number of co-infected virus were compared among severity groups.

Characteristics, Symptoms and Laboratory Data of PIV-4 positive patients

**Figure:**
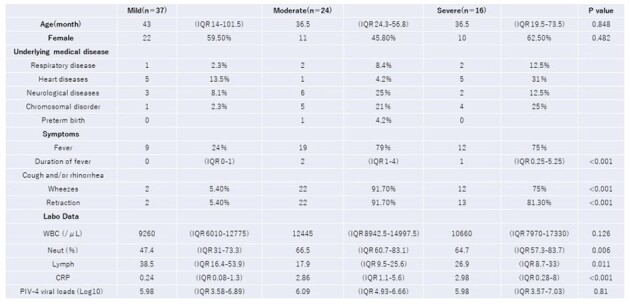

**Results:**

Nasal swab samples were collected from 2884 cases. PIV-4 was detected in 185 samples. As 108 cases were excluded for higher viral loads of other viruses, 77 cases were included for analysis. The severity of PIV-4 comprised 37 mild, 24 moderate, and 16 severe cases. The rate of lower respiratory tract infections in mild, moderate and severe cases were 8%, 96%, and 75%, respectively. There was only one PIV-4-related mortality at 28 days. Co-detection with rhinovirus/enterovirus was the most frequent. Median viral loads (log_10_ copies/mL) in mild, moderate and severe cases were 5.98 (IQR 3.58-6.89), 6.09 (IQR 4.93-6.66) and 5.98 (IQR 3.57-7.03), respectively. No significant correlation was found between viral load and severity, but severity correlated with the number of viral co-infections.

Treatment and Outcome of PIV-4 positive patients

**Figure:**
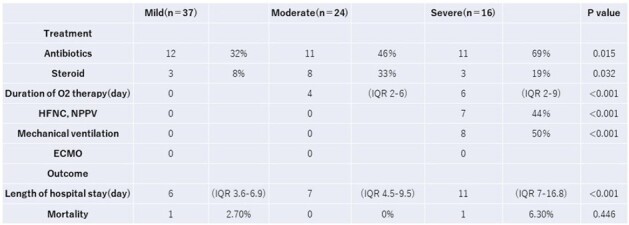

**Conclusion:**

Moderate or severe Pediatric PIV-4 infections primarily show as lower respiratory tract infections, with no relation between viral load and illness severity, though the number of co-infected virus may play a role.

Severity and co-infection viruses

**Figure:**
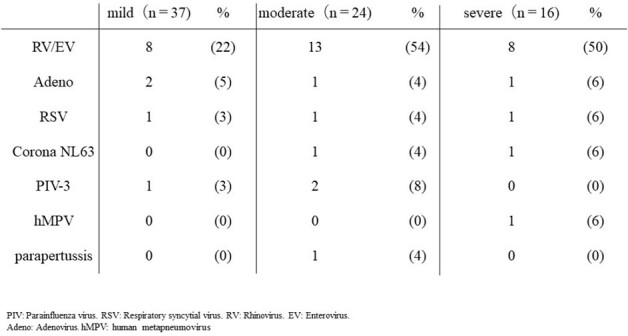

**Disclosures:**

**All Authors**: No reported disclosures

